# Individual income and race-associated differences in prostate cancer mortality in a statewide registry

**DOI:** 10.1093/jncics/pkaf074

**Published:** 2025-10-24

**Authors:** Alec Zhu, Stephen Rhodes, Bashir Al Hussein Al Awamlh, Randy A Vince, Nicholas Zaorsky, Daniel E Spratt, Camilo Arenas Gallo, Anyull Dayanna Bohorquez Caballero, Mollie Goldman, Jonathan E Shoag

**Affiliations:** Department of Urology, New York-Presbyterian Hospital, Weill Cornell Medicine, New York, NY, United States; Department of Urology, University Hospitals Cleveland Medical Center, Case Western Reserve University School of Medicine, Cleveland, OH, United States; Department of Urology, New York-Presbyterian Hospital, Weill Cornell Medicine, New York, NY, United States; Department of Urology, University Hospitals Cleveland Medical Center, Case Western Reserve University School of Medicine, Cleveland, OH, United States; Department of Radiation Oncology, University Hospitals Cleveland Medical Center, Case Western Reserve University School of Medicine, Cleveland, OH, United States; Department of Radiation Oncology, University Hospitals Cleveland Medical Center, Case Western Reserve University School of Medicine, Cleveland, OH, United States; Department of Urology, University Hospitals Cleveland Medical Center, Case Western Reserve University School of Medicine, Cleveland, OH, United States; Department of Urology, University Hospitals Cleveland Medical Center, Case Western Reserve University School of Medicine, Cleveland, OH, United States; Department of Urology, University Hospitals Cleveland Medical Center, Case Western Reserve University School of Medicine, Cleveland, OH, United States; Department of Urology, University Hospitals Cleveland Medical Center, Case Western Reserve University School of Medicine, Cleveland, OH, United States

## Abstract

**Background:**

Prior studies evaluating racial disparities in cancer outcomes used regional measures of deprivation when accounting for socioeconomic status, which lack granularity. We evaluated differences in prostate cancer mortality between Black and White men using individual home prices in addition to regional metrics to understand the impact of individual wealth on prostate cancer outcomes.

**Methods:**

Individuals diagnosed with prostate cancer between January 2004 and December 2016 in the Ohio Cancer Incidence Surveillance System were included. Individual home addresses were linked to the Area Deprivation Indices and home prices using data from an online real estate marketplace. Using inverse probability weighting to balance patient characteristics, we assessed differences in prostate cancer–specific mortality or other-cause mortality between Black and White men after accounting for clinical characteristics and social determinants of health (insurance, area deprivation, and home price).

**Results:**

We identified 70 660 (85%) White and 12 192 (15%) Black men with prostate cancer. Black race was associated with a higher risk of prostate cancer–specific mortality in models that adjusted for age and year at diagnosis (subdistribution hazard ratio = 1.45, 95% CI = 1.45 to 1.57) and with the addition of cancer variables (subdistribution hazard ratio = 1.16, 95% CI = 1.06 to 1.26). In models that incorporate social determinants of health, however, rates of prostate cancer–specific mortality and other-cause mortality were not statistically significantly higher for Black men (subdistribution hazard ratios = 1.10, 95% CI = 0.98 to 1.24, and 1.02, 95% CI = 0.95 to 1.09), respectively.

**Conclusions:**

After accounting for clinical characteristics and social determinants of health at the individual level, Black men were not at increased risk of prostate cancer mortality relative to White men.

## Introduction

In the United States, Black men are more likely to present with prostate cancer at a younger age and more advanced stages^1^ and have higher rates of prostate cancer incidence and cancer-specific mortality than White men.^2-4^ Disparities between Black and White men stem from a complex interplay of genetic predispositions, structural factors, and social determinants of health (SDOH).^5^ Men with higher socioeconomic status (SES) have better outcomes than men with lower SES, likely due to improved access to care, health literacy, and financial stability. The correlation between SDOH and health outcomes is further supported by studies that demonstrate the mitigation of disparities between Black and White men in equal-access settings.^6-9^

Prior studies evaluating the association between socioeconomic deprivation and cancer outcomes used regional measures of deprivation, such as the Area Deprivation Index (ADI), and income.^1-12^ At the most granular level, these metrics average data across census block groups, which comprise between 600 and 3000 individuals.^13^^,^^14^ Averaging data across many patients may be necessary given the limitations of most datasets, but such methods lead to loss of granularity that would otherwise be captured by individual-level data.^15^ Existing studies that use area-based SDOH may not fully capture the complex relationship between deprivation and outcomes.^16^ Recent work used a population-based dataset that includes individual-level (home prices) and area-based (ADI) markers of socioeconomic deprivation and demonstrated the substantial impact that higher levels of individual wealth (as measured by home price as a surrogate for income) have on mortality outcomes across multiple cancer types, including prostate cancer.^17^ Using this unique dataset, we sought to evaluate differences in prostate cancer-specific mortality between Black and White patients after accounting for SDOH at the individual level.

## Methods

### Study design and population

**Figure 1.**
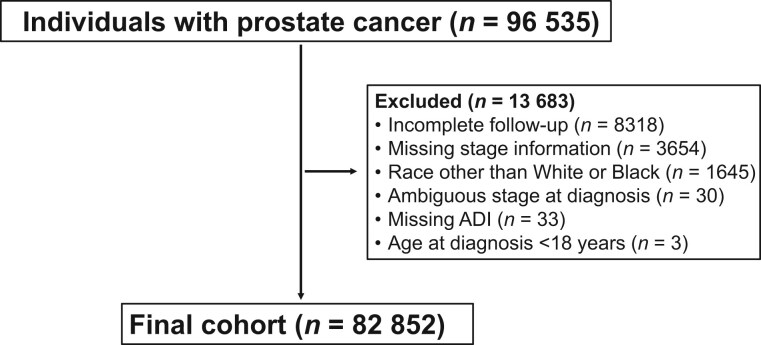
CONSORT diagram. Abbreviation: ADI = Area Deprivation Index.

This retrospective cohort study examined adult patients captured within the Ohio Cancer Incidence Surveillance System (OCISS)—a population-based registry that includes cancer incidence and outcomes for all residents of Ohio—who were diagnosed with prostate cancer between January 2004 and December 2016. Follow-up duration extended through 2018. Patients were excluded for having incomplete follow-up, being of a race other than Black or White, missing disease stage at diagnosis, or ADI (Figure 1). Baseline demographic data, including age, year of diagnosis, disease stage at diagnosis (localized, regional, or distant), insurance status, pretreatment prostate-specific antigen (PSA) level, and Gleason score, were captured. Prostate cancer site was identified based on the *International Classification of Diseases for Oncology, 3rd Edition*, codes (Table S1). We obtained PSA levels from Collaborative Stage Site-Specific Factor 1 and Gleason score from Collaborative Stage Site-Specific Factors 5, 6, 7, and 8 of the Surveillance, Epidemiology, and End Results Program (SEER) Registrar Staging Assistant.^18^ Cancer stage at diagnosis was separated into categories of localized, regional, or distant. This study was approved by the Ohio Department of Health (protocol No. 2019-5) and the Case Western Reserve University (protocol No. 20190455) institutional review boards.

### Income measurement

Individual home addresses at the time of diagnosis, obtained from the OCISS, were linked to home price estimates (Zestimate) from Zillow. The Zestimate uses public and user-submitted data, such as home details, location, property tax assessments, sales histories, and other homes sold in the area, to estimate home price, although its exact method is proprietary.^19^ Home price estimates in 2022 were used because historical estimates were not available. Although home prices may have shifted due to the global pandemic,^20^ our analysis reflects the assumption that the relative rank ordering of home prices is stable over time.^21^

Individual data were also linked to ADI, obtained from the 2019 American Community Survey, to measure socioeconomic deprivation. The ADI is a measure of economic and material deprivation consisting of 15 US Census variables, such as median family income, education distribution, and unemployment rate, and is determined at the census block group level.^13^^,^^22^ Increasing ADI values represent greater levels of socioeconomic deprivation.

### Outcomes

The primary outcome was cumulative incidence of prostate cancer–specific mortality, defined by the number of days from diagnosis to mortality. Prostate cancer–specific mortality was determined by SEER cause-of-death codes on death certificate reports for prostate cancer (Table S1). Additional outcomes were other-cause mortality, which included any cause of death not related to prostate cancer. Secondary analysis was performed to determine how White and Black race affects outcomes of age, stage, Gleason score, and PSA level at the time of prostate cancer diagnosis.

### Statistical analysis

We assessed differences in prostate cancer–specific mortality or other-cause mortality between White and Black patients with prostate cancer after adjusting for baseline characteristics, cancer stage, and SDOH variables (insurance, area deprivation, and home price estimate). Bivariate analysis was performed using Kruskal-Wallis or χ^2^ tests for continuous and categorical variables, respectively. Inverse probability weighting was used to balance various characteristics between White and Black patients before comparison.^23^ Several propensity score models were constructed using logistic regression to predict Black race: (1) age and year at diagnosis; (2) the prior factors plus stage at diagnosis, PSA level, and Gleason scores; (3) the prior factors plus insurance status, ADI, and home price estimate; and (4) year at diagnosis and SDOH variables only. Predicted probabilities were used to create inverse probability weights, and weights derived from these models were stabilized using the overall proportion of the sample within the White and Black cohorts. Balance between White and Black cohorts was assessed using the standardized mean difference before and after treatment weighting with the 4 propensity score models (Figure S1). Standardized mean difference values below 0.1 were taken to indicate good balance.

Cumulative incidence of prostate cancer–specific mortality and other-cause mortality were plotted between White and Black patients, and differences in 10-year incidence probabilities were calculated. Weighted Fine-Gray models with subdistribution hazard ratios (HRs) were used to analyze the competing risks of prostate cancer–specific mortality and other-cause mortality between Black and White men.

For the secondary analysis, we used varying weighted models to estimate the differences in age, PSA level at diagnosis, and probability of each diagnosis stage and Gleason score between Black and White patients using multinomial regression. Additional methods are included in the Supplementary Methods. All analyses were performed in R, version 4.2.1, statistical software (R Foundation for Statistical Computing). The *sociome, rms*, and *survival* packages were used to obtain ADI and perform survival analyses.^24-26^

## Results

We identified 96 535 men in the OCISS with prostate cancer; 13 683 (14%) men were excluded for lack of follow-up duration, missing disease stage information, race other than White or Black, unknown disease stage at diagnosis, missing ADI, or age diagnosis under 18 years (Figure 1). Analysis was performed on the final cohort of 82 852 patients; 70 660 (85%) of patients were White and 12 192 (15%) of patients were Black.

### Baseline characteristics

**Figure 2.**
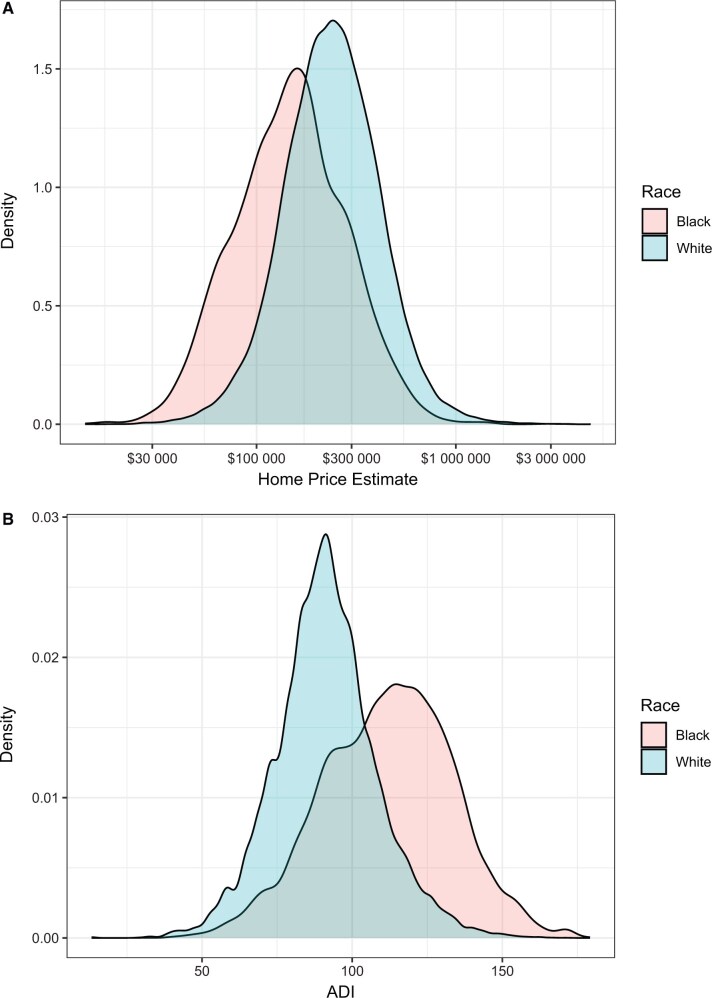
Density distribution of patients according to home price estimate (**A**) or Area Deprivation Index (ADI) (**B**), by race.

Baseline demographics, clinical characteristics, and prostate cancer mortality outcomes of patients by race are shown in Table 1. Median PSA values were higher in Black patients (7.5 vs 6.3, *P* < .001), and a higher proportion of Black men than White men had high-risk prostate cancer (Gleason score 8-10; *P* < .001). There was wide variation in estimated home prices and ADI across the 2 cohorts, but Black men tended to have lower estimated home prices and higher ADI than White men (Figure 2). Median home prices were $238 100 (IQR, $165 700-$341 600) for White patients and $149 000 (IQR, $95 375-$226 725) for Black patients (*P* < .001) (Table 1). Median ADI was lower for White patients than for Black patients (90.9 vs 113, *P* < .001). Although 32% of the men were missing home price values, Black men were more likely to have missing home prices than White men (46% vs 30%). In addition, 12% of Black patients and 16% of White patients were missing PSA data (Table S2).

**Table 1.** Baseline demographic and clinical characteristics.

Characteristic	White	Black	*P*
	***n* = 70** **660**	***n* = 12** **192**	
Age, median (IQR), y	66 (60-73)	64 (58-70)	<.001
Diagnosis year, median (IQR)	2010 (2007-2013)	2010 (2007-2013)	<.001
Pretreatment PSA level, median (IQR), µg/L	6.3 (4.6-10.8)	7.5 (4.9-16.3)	<.001
Home price estimate, median (IQR), $	238 100 (165 700-341 600)	149 000 (95 375-226 725)	<.001
ADI, median (IQR), units	91 (81-101)	113 (97-127)	<.001
Follow-up time, median (IQR), d	1928 (879-3004)	1776 (796-2862)	<.001
Hispanic ethnicity, No. (%)			<.001
Yes	460 (0.65)	33 (0.27)	
No	65 898 (93.00)	11 429 (94.00)	
Unknown	4302 (6.10)	730 (6.00)	
Gleason score, No. (%)			<.001
≤6	26 799 (38.0)	4139 (34.0)	
7	25 930 (37.0)	4782 (39.0)	
8	6323 (8.9)	1231 (10.0)	
9-10	6095 (8.6)	1107 (9.1)	
Unknown	5513 (7.8)	993 (7.7)	
Stage at diagnosis, No. (%)			<.001
Localized	58 859 (83.0)	10 128 (83.0)	
Regional	8340 (12.0)	1230 (10.0)	
Distant	3461 (4.9)	834 (6.8)	
Insurance group, No. (%)			<.001
Commercial	28 314 (40.0)	4672 (38.0)	
Medicare	34 259 (48.0)	4942 (41.0)	
Medicaid/public health	1229 (1.7)	1007 (8.3)	
Military/US Department of Veterans Affairs	1363 (1.9)	545 (4.5)	
Not Insured	913 (1.3)	403 (3.3)	
Unknown	4582 (6.5)	623 (5.1)	
Mortality, No. (%)			<.001
Prostate cancer specific	3787 (5.4)	774 (6.3)	
Other cause	12 359 (17)	2099 (17)	

Abbreviations: ADI = Area Deprivation Index; PSA = prostate-specific antigen.

Several propensity score models were generated with varying degrees of weighting for the primary analysis of prostate cancer–specific and other-cause mortality outcomes (Figure S1). In the fully weighted propensity score model (accounting for age, year, stage, PSA level, Gleason score, insurance status, ADI, and home price), all variables were well balanced, with an absolute standardized mean difference below 0.1 (Figure S1, D). Separate propensity score models were also generated for the analysis of age, stage, Gleason score, and PSA level at diagnosis, and all included variables were well balanced (standardized mean difference < 0.1) in the fully weighted models (Figure S1, G-I).

### Primary outcomes

**Figure 3.**
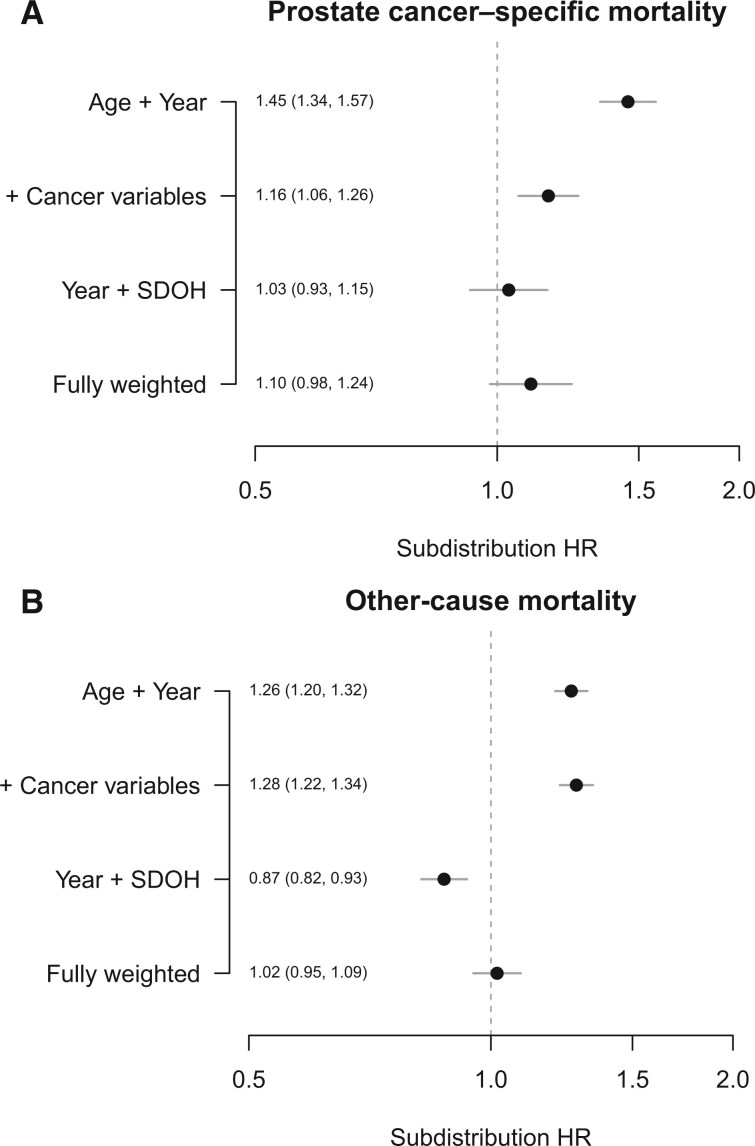
Weighted Fine-Gray competing risks of prostate cancer–specific mortality and other-cause mortality, with incorporation of varying demographic and clinical factors. Subdistribution hazard ratios are demonstrated for Black vs White race (ie, a subdistribution HR > 1 means higher risk for Black vs White men). Abbreviations: HR = hazard ratio; SDOH = social determinants of health.

Competing risks of prostate cancer–specific mortality and other-cause mortality were assessed using weighted Fine-Gray models (Figure 3). With the incorporation of clinical characteristics alone, subdistribution hazard ratios demonstrated a higher risk of prostate cancer–specific mortality for Black race when adjusting for age and year at diagnosis (subdistribution HR = 1.45, 95% CI = 1.34 to 1.57) and with the addition of cancer variables (subdistribution HR = 1.16, 95% CI = 1.06 to 1.26). In the fully weighted model that included SDOH (insurance status, ADI, and home price), however, the risk of prostate cancer–specific mortality was not statistically significantly higher for Black men (subdistribution HR = 1.10, 95% CI = 0.98 to 1.24). Even when including only year at diagnosis and SDOH variables, Black race was not associated with higher risk of prostate cancer–specific mortality (subdistribution HR = 1.03, 95% CI = 0.93 to 1.15). Similar results were found for the other-cause mortality outcomes; in the fully weighted model, the risk of other-cause mortality was not statistically significantly higher for Black men (subdistribution HR = 1.02, 95% CI = 0.95 to 1.09).

Stratified analyses evaluating risk of mortality between White and Black patients were performed with weighted models, excluding home price or ADI (Figure S2). The risk of prostate cancer–specific mortality was not higher in Black men when omitting home price (subdistribution HR = 1.11, 95% CI = 0.99 to 1.25) but was higher when omitting ADI (subdistribution HR = 1.13, 95% CI = 1.02 to 1.25). The risk of other-cause mortality was statistically significantly higher in Black men when omitting home price (subdistribution HR = 1.26, 95% CI = 1.20 to 1.32) and when omitting ADI (subdistribution HR = 1.12, 95% CI = 1.06 to 1.19).

When evaluating the risk of mortality over different diagnosis years, Black race was associated with higher prostate cancer–specific mortality between 2004 and 2006, but Black race was not associated with higher risk of prostate cancer–specific mortality between the other diagnosis years (Figure S3).

**Figure 4.**
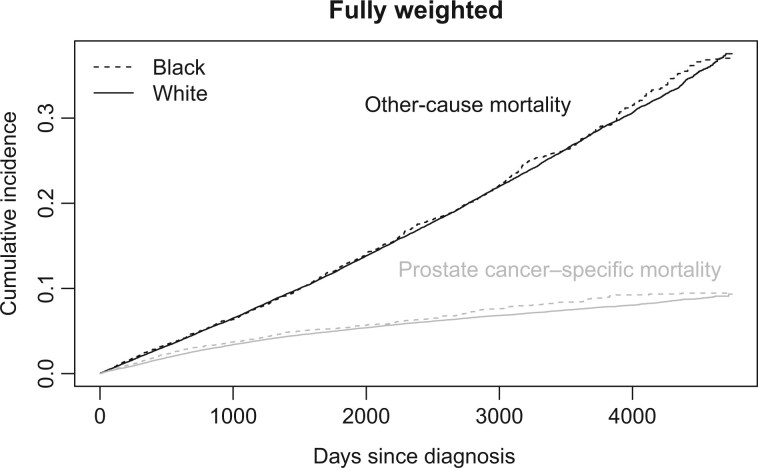
Cumulative incidence of prostate-cancer specific mortality and other-cause mortality after inverse probability weighting.

Using the fully weighted data, the 10-year incidence probabilities were 0.008 (95% CI = ‒0.002 to 0.019) higher for prostate cancer–specific mortality and 0.001 (95% CI = ‒0.018 to 0.020) higher for other-cause mortality in Black vs White patients (Figure 4).

### Secondary outcomes

**Figure 5.**
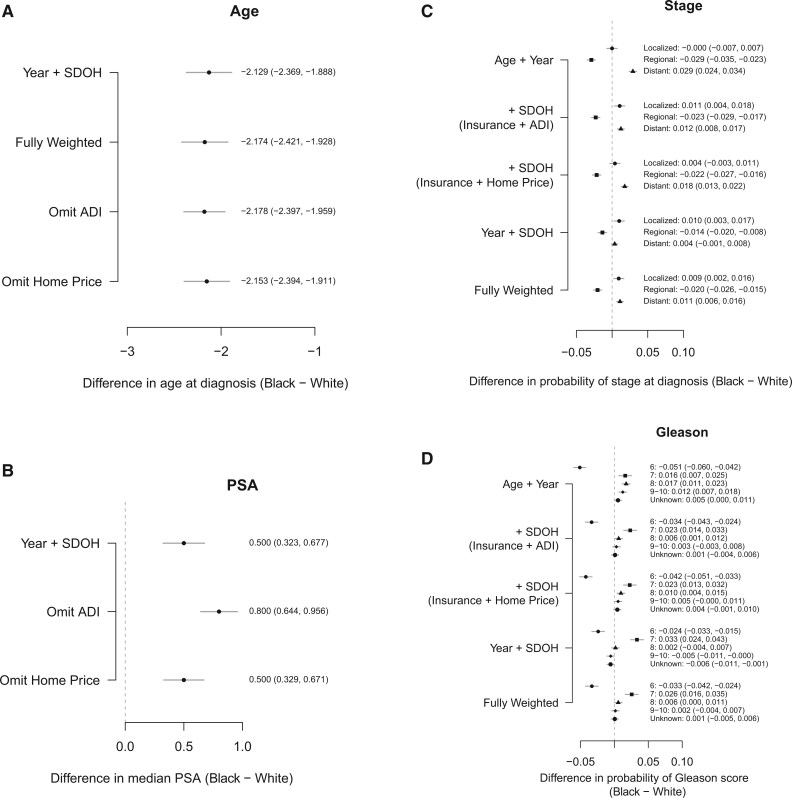
Estimated differences in the age (**A**), median PSA (**B**), and probability of stage (**C**) and Gleason score (**D**) at diagnosis between Black and White patients. Abbreviations: ADI = Area Deprivation Index; PSA = prostate-specific antigen; SDOH = social determinants of health.

In weighted analyses of age at diagnosis, Black men were on average diagnosed with prostate cancer at a younger age (by about 2 years) (Figure 5, A). Black men also had higher median PSA values (0.500, 95% CI = 0.323 to 0.677) than White men (Figure 5, B).

For the analysis of stage at diagnosis (Figure 5, C), Black men had a 0.9% (95% CI = 0.2% to 1.6%) higher probability of being diagnosed at the localized stage and 1.1% (95% CI = 0.6% to 1.6%) higher probability of being diagnosed at the distant stage in the fully weighted model. Black men also had a lower probability of being diagnosed at the regional stage (‒0.2%, 95% CI = ‒2.6% to ‒1.5%]). When evaluating Gleason score at diagnosis, Black men had a lower probability of being diagnosed with Gleason 6 and a higher probability of being diagnosed with Gleason 7 cancer compared with White men. There were no statistically significant differences, however, in probability of diagnosis of Gleason 8, 9, or 10 cancers (Figure 5, D).

## Discussion

Using a statewide cancer registry, we evaluated differences in mortality outcomes between Black and White men with prostate cancer. After accounting for clinical characteristics alone, Black race was associated with a higher risk of both prostate cancer–specific mortality and other-cause mortality compared with White race. After accounting for regional and individual-level SDOH, however, Black race was not associated with a higher risk of prostate cancer–specific mortality or other-cause mortality. Although Black patients tended to be diagnosed at an earlier age and at a higher median PSA value, Black men were not more likely than White men to be diagnosed with high-risk prostate cancer.

Racial disparities in prostate cancer mortality stem from factors that span the cancer continuum, from screening to diagnosis to treatment. Addressing racial inequities in prostate cancer outcomes requires understanding specific driving factors that contribute to the excess mortality seen in Black men. For instance, Black men are more likely to be diagnosed with prostate cancer at younger ages and at a more advanced stages of disease,^27^ which may account for lower cancer-specific survival rates among Black men.^28^^,^^29^ In addition, using a prostate cancer model across the SEER database, Gulati et al.^30^ found that increased incidence, more aggressive tumor biology, and worse baseline cancer-specific survival in Black men accounted for 38%, 34%, and 30% of the disparity in mortality, respectively. Consistent with this work, we found that Black men have increased prostate cancer–specific mortality and other-cause mortality relative to White men when accounting for prostate cancer factors such as age, stage at diagnosis, PSA value, and Gleason score. Furthermore, Black men are diagnosed at earlier ages and are more likely to be diagnosed at a distant stage than White men.

Although race has been readily used in the risk stratification and management of patients with prostate cancer, there is a growing movement to reconsider the utility of racial classifications. Vyas et al.^31^ questioned the role of race in clinical algorithms across multiple medical disciplines given its potential to inappropriately simplify the interpretation of outcomes. More specifically, experts have evaluated the role of race-based prostate cancer screening recommendations and highlighted their risk in obscuring the impact of socioeconomic factors.^32^ The disparities in prostate cancer mortality between Black and White men likely reflect differences in both screening and treatment effectiveness between these 2 groups, and SDOH plays an important role in how patients can access health services. Studies have demonstrated the substantial impact of SES on prostate cancer outcomes, whereby men of higher SES have improved outcomes compared with men of lower status.^33-35^ On a regional level, men living in the most deprived neighborhood had an increased risk of prostate cancer mortality compared with men living in the least deprived neighborhoods.^10^^,^^36-39^ Ellis et al.^28^ found that cancer stage at diagnosis accounted for nearly one-quarter of the excess mortality risk in Black men, while neighborhood SES accounted for 7% of this disparity. Various studies examining SES, however, including those studying income,^12^ used neighborhood-level factors at the census tract. Regional measures of SES may not account for individual differences in SES.^16^ For example, Afshar et al.^40^ showed that individuals living in more disadvantaged areas had lower 5-year survival across 21 cancers, including prostate cancer, but there was a lack of individual-level data to provide greater granularity on patients’ SES.

For the first time, our analysis of SDOH used home price estimates—a proxy for individual income—as well as ADI to quantify deprivation at the individual and census block levels.^13^^,^^22^ Although fully adjusted models incorporating both home price and ADI showed no statistically significant differences in prostate cancer–specific or other-cause mortality between Black and White men, models excluding home price or ADI demonstrated greater other-cause mortality but not prostate cancer–specific mortality in Black men. Our prior work from OCISS demonstrated that survival models that incorporated home price provided stronger predictive value than those using ADI, and models using both home price and ADI were superior to those that used only 1 metric.^17^ These results suggest that home price and ADI may be capturing overlapping and distinct aspects of SDOH, and these factors more profoundly affect estimates of other-cause mortality.

Our results are consistent with prior studies that demonstrated that mortality outcomes in Black and White men are similar in “equal-access” settings.^6-9^ For example, among men with nonmetastatic prostate cancer, Dess et al. demonstrated that Black race was associated with increased risk of prostate cancer–specific mortality within the SEER registry, but Black race was not associated with increased mortality risk in a US Department of Veterans Affairs cohort and was associated with reduced mortality risk in a randomized clinical trial cohort.^9^ Race-based differences in prostate cancer–specific mortality from SEER may be explained by residual confounding of race with SDOH. Variables of SES in SEER are taken at the census tract level,^41^^,^^42^ whereas our results uniquely provide individual-level estimations of income. Although Black men had worse cancer mortality outcomes than White men after accounting for clinical characteristics such as age, stage at diagnosis, PSA level, and Gleason score, these disparities disappeared after accounting for insurance status, ADI, and individual home price. In addition, even without accounting for clinical characteristics (only year at diagnosis and SDOH), Black men did not exhibit worse prostate cancer–specific mortality than White men.

Our study must be considered within the context of its limitations. First, the study cohort included men with missing home price and PSA values, which may introduce bias with respect to race. Therefore, we used a missing indicator approach to model home price and PSA value; missing observations were replaced with the average of the observed, and an additional binary variable was included indicating whether home price or PSA level was observed. Second, home values based on Zestimate were used as a surrogate marker for individual income, but this approach may not completely reflect one’s overall wealth. Assets beyond home ownership, such as retirement plans and businesses, are not accounted for, and individuals who rent homes would have a less accurate representation of their income using our analysis. Nevertheless, home equity is the most substantial contributor to individual wealth for individuals with net worths up to $1 million,^43^ which would include most individuals. Finally, comorbidity data, which may confound the relationship between race and survival, were unavailable and cannot be accounted for in our analysis.

In a statewide registry (OCISS), Black men have worse prostate cancer–specific mortality than White men, after accounting for clinical characteristics such as stage at diagnosis, PSA level, and Gleason score. After including social factors such as insurance status, ADI, and income, however, Black and White men have similar prostate cancer–specific mortality. By incorporating home price as a surrogate of income, our study is the first to use an individual-level metric of SES to evaluate racial disparities in prostate cancer outcomes. These results demonstrate that racial parity in cancer outcomes is possible, and addressing modifiable factors, such as increased screening, may be an important component in alleviating racial disparities in prostate cancer.

## Supplementary Material

pkaf074_Supplementary_Data

## Data Availability

The data used in this study are not available because they were obtained under license from the OCISS.
