# Supplementary material for: Individual income and race-associated differences in prostate cancer mortality in a statewide registry
Source: JNCI Cancer Spectr. 2025 Oct 24;9(5):pkaf074. doi: 10.1093/jncics/pkaf074 (PMC12571109; doi:10.1093/jncics/pkaf074)

SUPPLEMENTARY MATERIAL

Table of Contents

Supplementary Methods **2**

Supplementary Tables **3-4**

Table S1 3

Table S2 4

Supplementary Figures **5-7**

Figure S1 5

Figure S2 6

Figure S3 7

Supplementary Methods

ADI and home price were modeled via natural cubic splines with 6 degrees of freedom (5 knots). We used a missing indicator approach to model home price and PSA where missing observations were replaced with the average of the observed, and an additional binary variable was included in the propensity score model denoting whether home price was observed or not. Thus, White and Black patients were matched on home price values as well as the propensity to have missing home price information.

To evaluate differences in mortality after incorporating individual (home price) or regional (ADI) measures of deprivation, stratified analyses were performed by constructing propensity models excluding home price or ADI. Additionally, because the difference in PCSM between Black and White men may have waned or exacerbated over time, a stratified comparison was also performed evaluating the risk of mortality over different diagnosis years.

For the secondary analysis, we used the same weighted model (including age, year at diagnosis, and SDH) to estimate the differences in probability of each stage at diagnosis and Gleason score between Black and White patients via multinomial regression. To estimate differences in PSA at diagnosis, we omitted those with missing PSA values and estimated race differences in median PSA via quantile regression. For the weighted analysis of differences in the age at diagnosis, a propensity score model was generated incorporating year, stage at diagnosis, insurance status, ADI, and home price. Multinomial models were generated via the 'nnet' package and contrasts and robust standard errors were calculated using the 'marginaleffects' package.

Table S1. The International Classification of Diseases for Oncology Third Edition (ICD-O-3) site code used to identify men with prostate cancer diagnoses. The Surveillance, Epidemiology, and End Results (SEER) Cause-of-death codes were used to determine cancer-specific survival outcomes.

Site	SEER Cause-of-death
C619	C61, 185, 600, 601, 602, 603, 604, 605, 606, 607, 608, N40, N41, N42, N43, N44, N45, N46, N47, N48, N49, N50

Table S2. Number of White or Black subjects missing demographic and clinical characteristics

N (%)	White n=70,660	Black n=12,192
Age	0 (0)	0 (0)
Diagnosis year	0 (0)	0 (0)
Hispanic ethnicity	0 (0)	0 (0)
Insurance group	0 (0)	0 (0)
Home price estimate	20,971 (29.7)	5,572 (45.7)
AD	0 (0)	0 (0)
Pretreatment PSA	11,536 (16.3)	1,465 (12.0)
Gleason score	0 (0)	0 (0)
Stage at diagnosis	0 (0)	0 (0)
Follow-up time	0 (0)	0 (0)
Prostate-cancer specific or other-cause mortality	0 (0)	0 (0)

Figure S1. Absolute SMD before and after treatment weighting for propensity models analyzing prostate cancer specific mortality or other cause mortality incorporating age and year at diagnosis (A), age, year and stage at diagnosis (B), year and social determinants of health (SDH) variables (C), all variables (D), all variables except home price estimate (E), and all variables except ADI (F). A fully weighted model including year, stage, SDH variables was used for analyzing age at diagnosis (G). A weighted model including age, year, and SDH variables was used for analyzing stage and Gleason score at diagnosis (H). A weighted model including year and SDH variables was used for analyzing PSA (I).

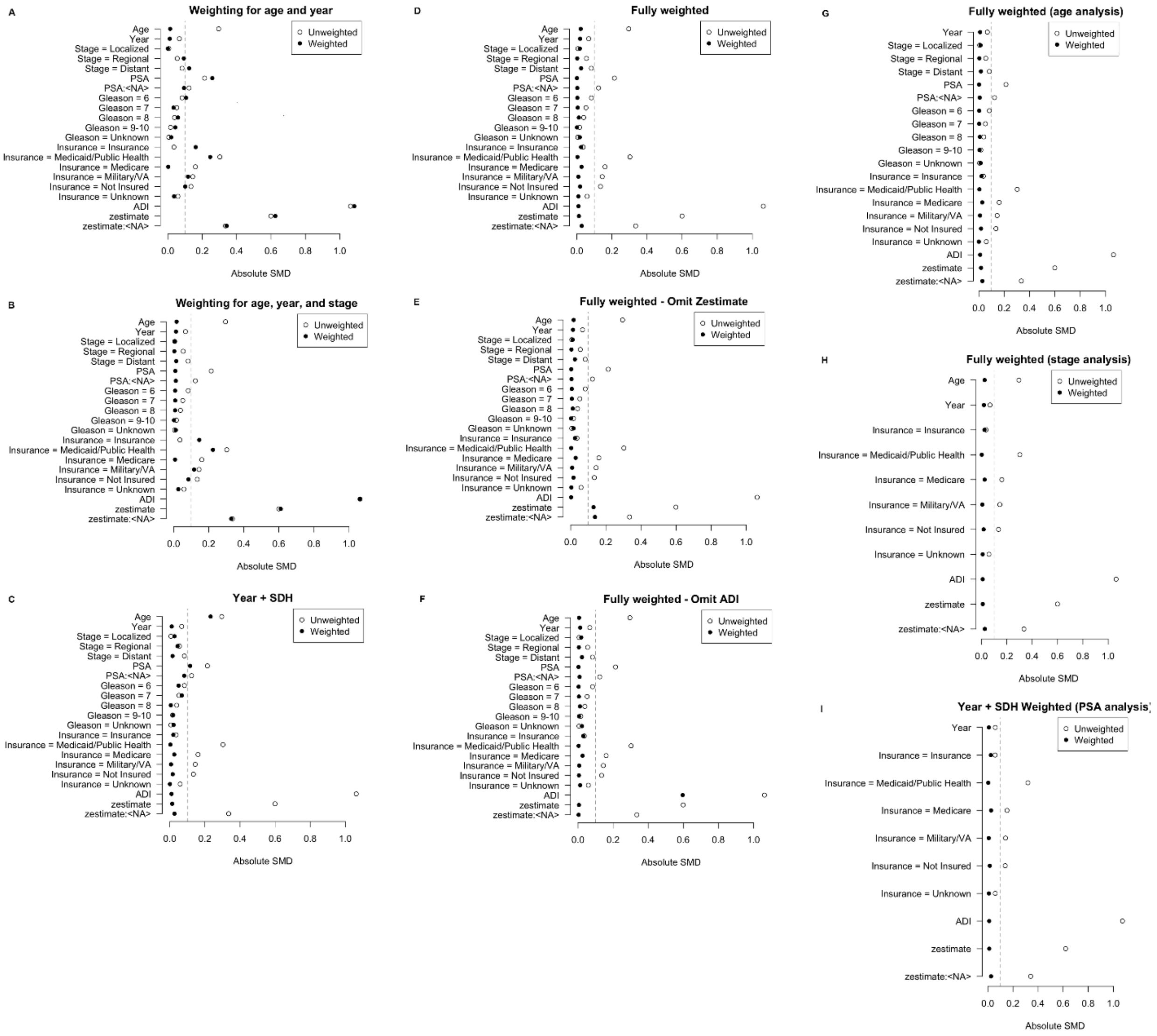

Figure S2. Weighted Fine-Gray competing risks of prostate cancer-specific mortality (A) and other-cause mortality (B) in models omitting home price estimate or ADI.

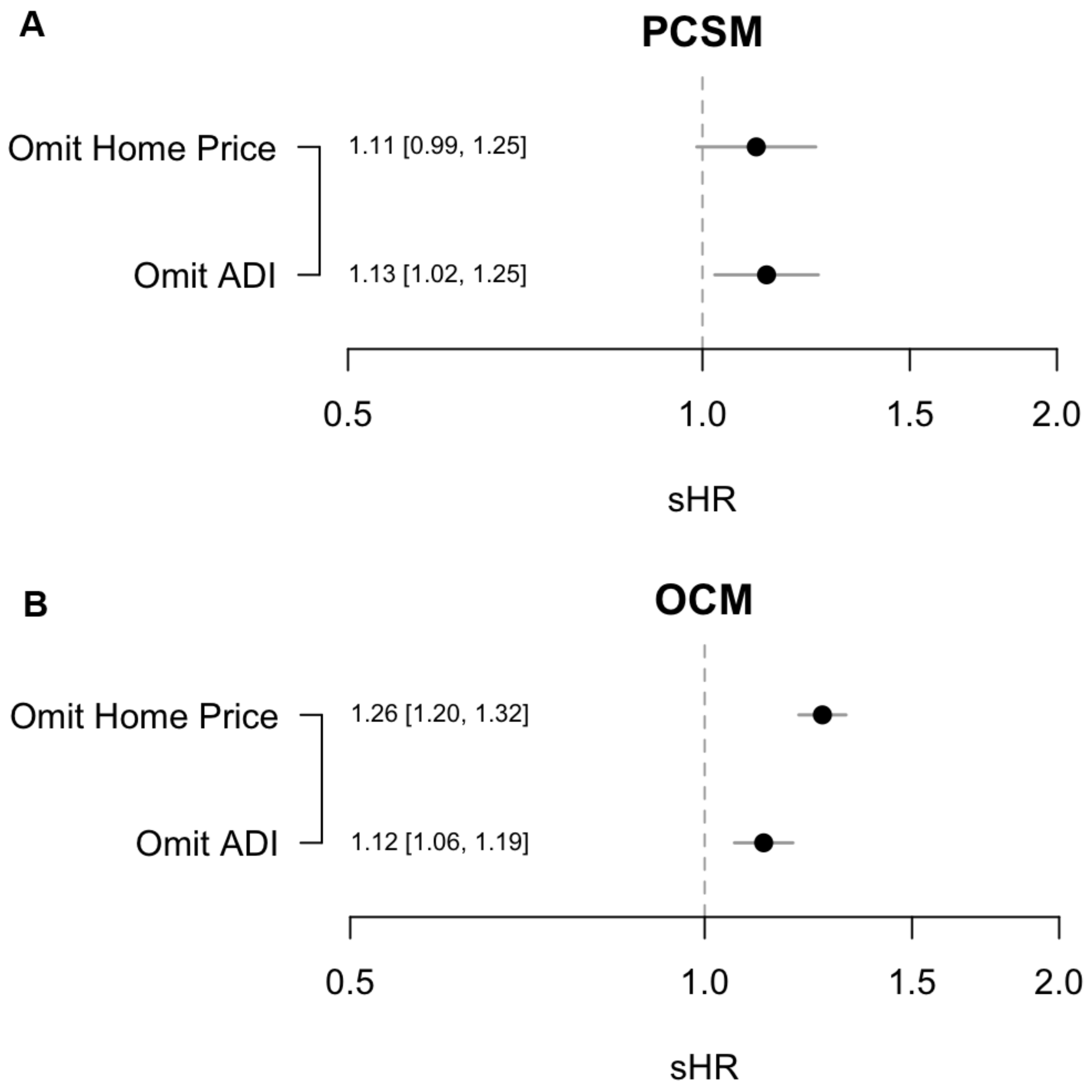

Figure S3. Difference in prostate-cancer mortality and other-cause mortality averaged over different diagnosis years.

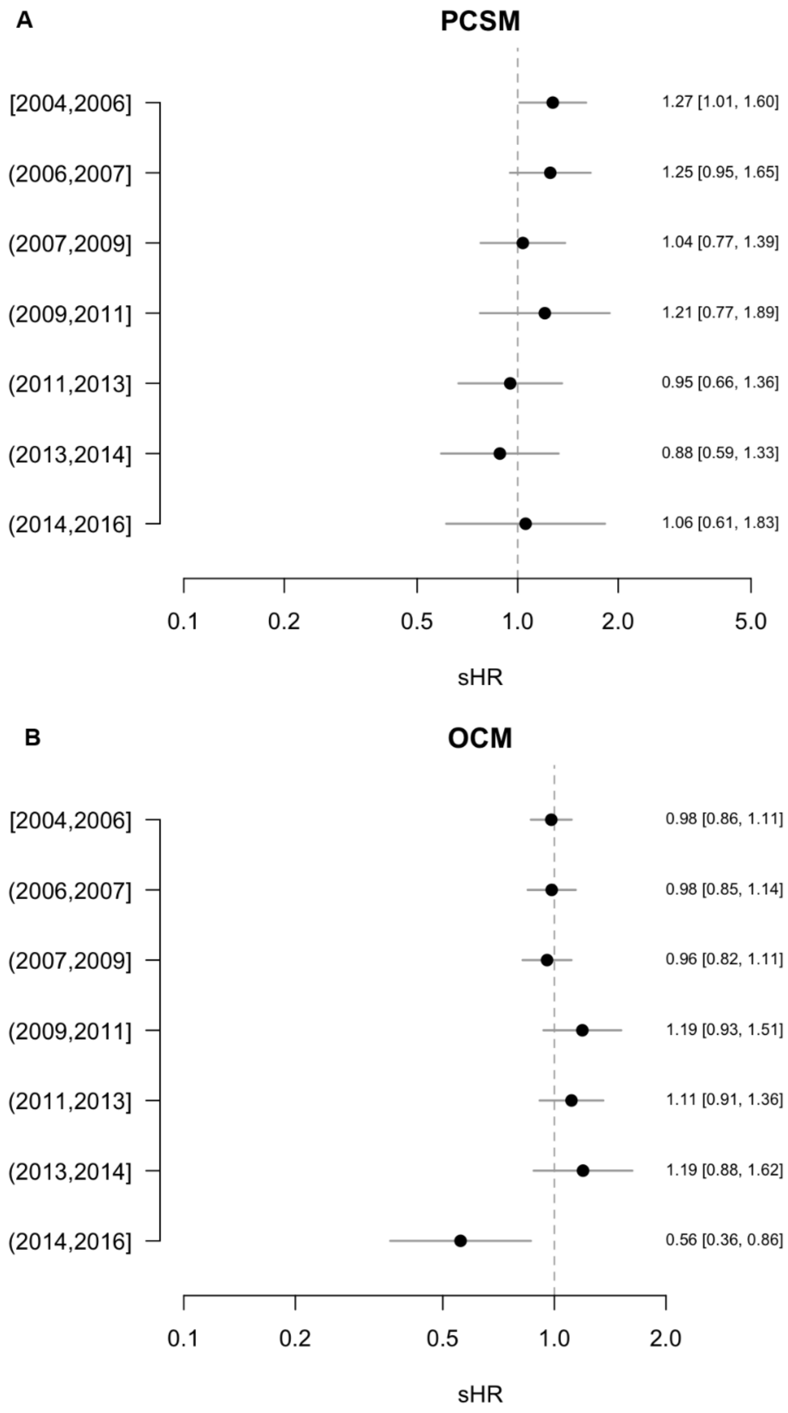

Supplement: pkaf074_Supplementary_Data [file pkaf074_supplementary_data.zip › Revisions_Supplemental Materials_JNCICS.pdf]
